# Orbital Apex Syndrome Secondary to SMARCB1-Deficient Invasive Sinonasal Carcinoma

**DOI:** 10.7759/cureus.31017

**Published:** 2022-11-02

**Authors:** Denzel Massey, Mathew Saab

**Affiliations:** 1 Emergency Medicine, Madigan Army Medical Center, Joint Base Lewis-McChord, USA

**Keywords:** facial swelling, optic nerve dysfunction, opthalmoplegia, smarcb1, sinonasal tumor, orbital apex syndrome

## Abstract

Orbital apex syndrome (OAS) is a clinical entity defined by ophthalmoplegia and optic nerve dysfunction due to local disruption of the orbital apex. The causes of OAS are extensive and include infectious, inflammatory, traumatic, iatrogenic, and neoplastic conditions. Thus, appropriate management is dependent on an accurate and timely diagnosis of the underlying etiology. We present a case of a 58-year-old female who presented to the emergency department with ophthalmoplegia of subacute onset and diminished visual acuity in the setting of two weeks of headache, ocular pain, and facial swelling. She was ultimately diagnosed with OAS and admitted to the hospital for five days for further evaluation. She was found to have an incurable primary SMARCB1-deficient sinonasal carcinoma with an invasion of her orbital apex. A multidisciplinary management approach involving chemotherapy, radiation, and surgical intervention was performed, and the patient responded well. Nearly two years after her diagnosis, she continues to have stable residual carcinoma without evidence of recurrence or metastatic disease. Her visual acuity has returned to normal limits, and her oculomotor function has returned to near-normal levels.

## Introduction

The human orbit is a pyramidal-shaped structure composed of four facial bones and three cranial bones. The apex is found at the posterior aspect of the orbit and is formed by the confluence of the four orbital walls [1]. Within this structure lies the optic canal, which contains the optic nerve and the ophthalmic artery, in addition to the superior orbital fissure, which lies lateral to the optic canal. The superior orbital fissure can be divided into superior, middle, and inferior portions defined according to their relation to the common tendinous ring [1]. The superior portion transmits the lacrimal nerve, frontal nerve, trochlear nerve, a superior branch of the ophthalmic vein, and recurrent meningeal artery [1]. The middle portion transmits the nasociliary nerve, abducens nerve, and the superior and inferior branches of the oculomotor nerve [1]. The inferior portion transmits the inferior branch of the ophthalmic vein [1].

Orbital apex syndrome (OAS) is defined by the optic nerve, oculomotor nerve, trochlear nerve, abducens nerve, and first division of the trigeminal nerve dysfunction secondary to violation of the orbital apex [2]. Clinical manifestations of OAS include vision loss, ophthalmoplegia, afferent pupillary defect, loss of corneal reflex, proptosis, periorbital pain, or hypoesthesia [1].

This syndrome can be caused by numerous etiologies including inflammatory, infectious, neoplastic, traumatic, iatrogenic, and vascular pathologies [1,2]. As a result, the management of OAS will vary from one patient to another and is dependent on the underlying etiology. Below, we present a case of OAS in a female patient secondary to an invasive sinonasal neoplasm.

## Case presentation

**Figure 1:**
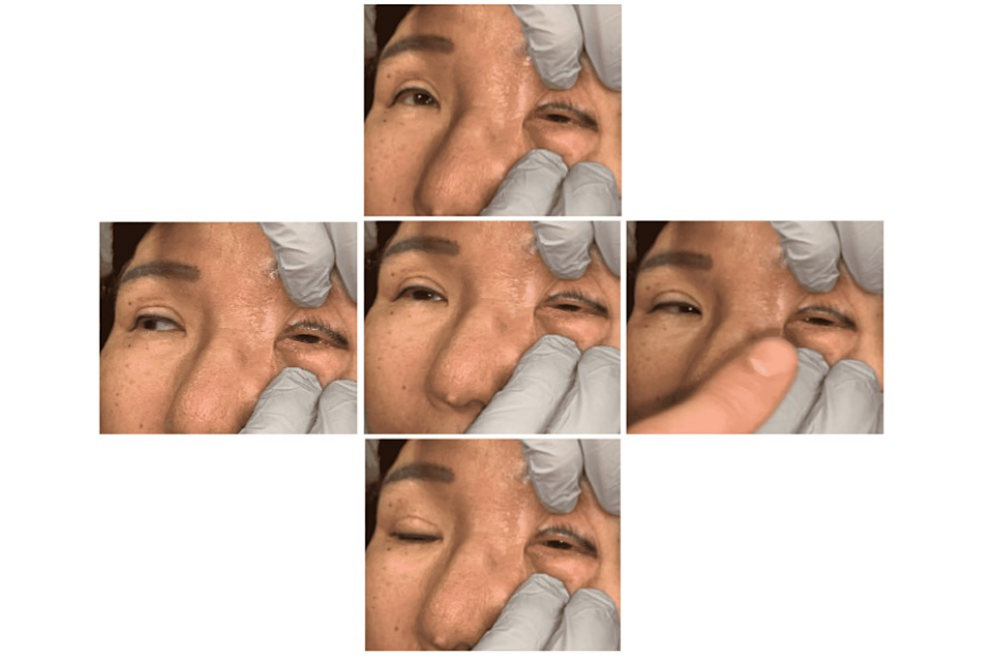
Patient demonstrating primary position, leftward, rightward, upward, and downward gaze. Note ophthalmoplegia of the left eye.

A 58-year-old female with a medical history of longstanding type 2 diabetes and a 30-year history of tobacco use presented to the emergency department (ED) with chief complaints of headache, left eye pain with vision impairment and left facial swelling. She reported that she was currently being evaluated by both her primary care physician and an otolaryngologist for recurrent episodes of epistaxis associated with chronic sinus pain, rhinorrhea, and nasal congestion, which had been occurring for the previous nine months. Over this period, she had a presumed diagnosis of chronic sinusitis and had been treated with antihistamines, cautery, and antibiotic courses of doxycycline and amoxicillin/clavulanic acid without any improvement of her symptoms. Two weeks before this presentation, she developed a gradual onset frontal headache with intermittent radiation to the occiput in addition to progressively worsening left-sided facial swelling, left periorbital swelling, and left eye mucopurulent discharge. Her clinical examination was notable for left periorbital swelling and ptosis with ophthalmoplegia as she had motility limitation with upward, downward, and lateral gazes of her left eye, and she reported pain with extraocular muscle activation (Figure 1).

**Figure 2:**
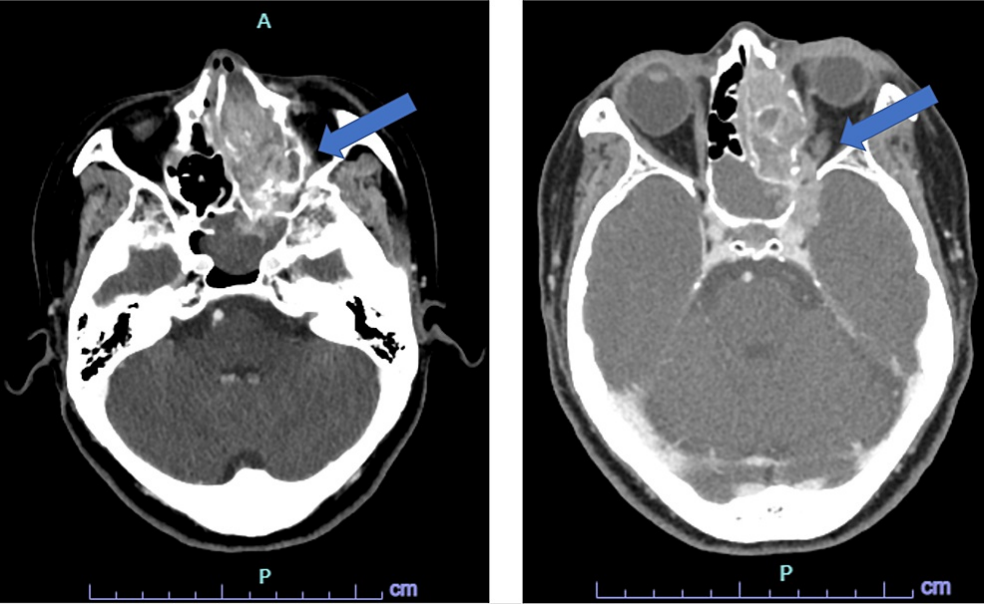
Contrast-enhanced CT imaging depicting opacification of left paranasal sinuses extending into cranium (arrow) with severe osseous erosion.

Her laboratory workup was notable for an elevated serum lactic acid level and anion gap metabolic acidosis, but white blood cell count, erythrocyte sedimentation rate, and C-reactive protein levels were within normal limits. In the ED, the patient was treated with a two-liter bolus of isotonic crystalloid, intravenous (IV) morphine and acetaminophen, and IV ceftriaxone after blood cultures were obtained. At this time, the differential diagnosis included acute sinusitis, orbital cellulitis, and cavernous sinus thrombosis, so a computed tomography (CT) of her brain, orbits and sinuses with intravenous (IV) contrast was ordered. The CT imaging showed extensive osseous erosion of the left medial orbit with opacification extending from the left paranasal sinuses through the superior orbital fissure and into the middle cranial fossa (Figure 2). There was also fat stranding of the periorbital tissues.

Initial antimicrobial coverage was quickly broadened to vancomycin, cefepime, and metronidazole upon review of the CT images. The patient was evaluated by an ophthalmology consultant in the ED who confirmed CN III, IV, and VI dysfunctions but also noted decreased visual acuity, an afferent pupillary defect, and increased intraocular pressure concerning CN II compression as well. The constellation of these findings confirmed the diagnosis of Orbital Apex Syndrome, and recommendations were made to initiate IV steroids, consider the urgent surgical evaluation, and further workup for an underlying infectious, inflammatory, or neoplastic etiology. An otolaryngology consultant performed a bedside nasal endoscopy with findings of a mass obstructing the left anterior nasal cavity. The patient was subsequently admitted to a medical/surgery ward for further evaluation.

During the hospital course, the patient was maintained on broad-spectrum antibiotics and IV dexamethasone. MRI and MRV imaging demonstrated an infiltrative lesion involving the left nasal cavity, pterygopalatine fossa, superior orbital fissure, and cavernous sinus surrounding the left internal carotid artery. There was no reported cavernous sinus thrombosis. Neurosurgery, otolaryngology, ophthalmology, and infectious disease teams were consulted, and after reviewing the imaging and performing functional endoscopic sinus surgery to obtain a biopsy of the lesion, it was determined that the patient had the unresectable disease and further surgical intervention was not warranted at that time. Serial ophthalmologic exams revealed progressively worsening visual acuity despite improvement in her extraocular movements and stable intraocular pressures. On hospital day five, tissue pathology via immunohistochemical techniques, confirmed SMARCB1-deficient sinonasal carcinoma, a rare aggressive type of malignancy. Investigations for an alternative infectious, autoimmune, or inflammatory disorder were unrevealing, so the antibiotics were discontinued, and the patient was discharged with an oral prednisone taper and close follow-up with oncology.

**Figure 3:**
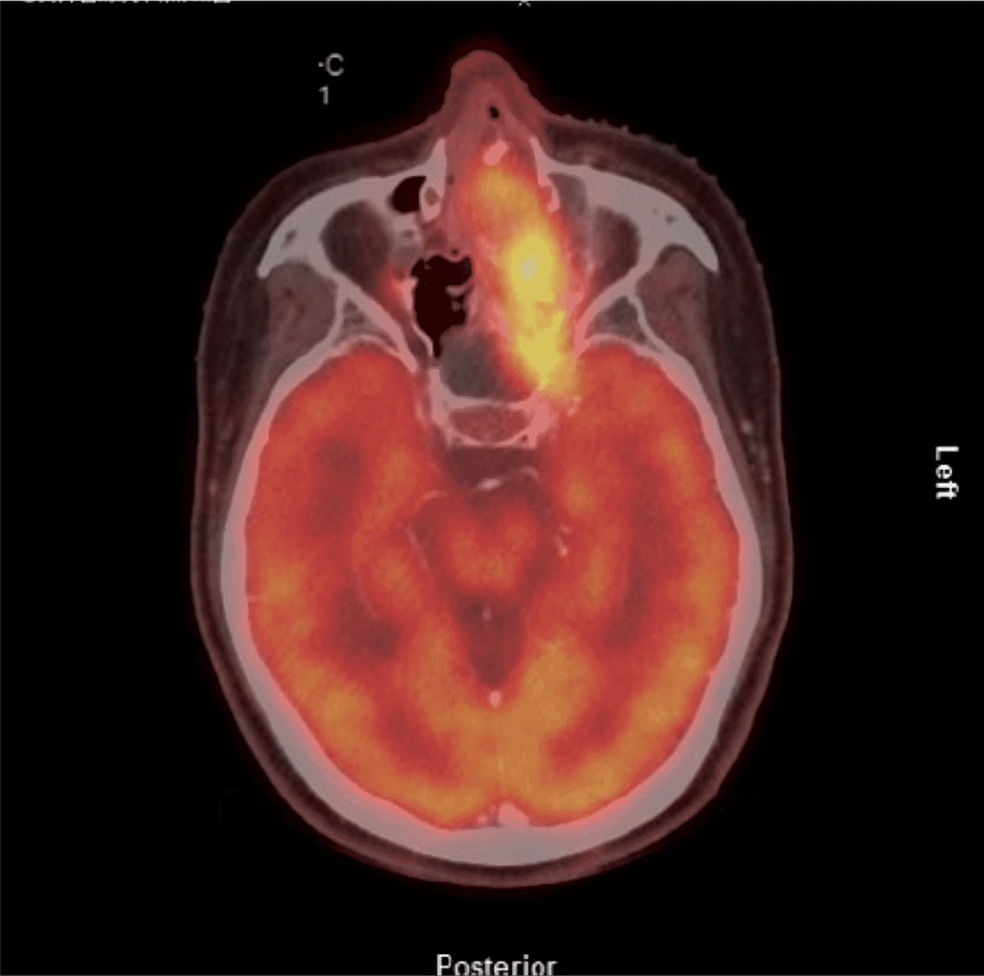
Positron emission tomography (PET) CT demonstrating a significant FDG uptake of metabolically active lesion.

On post-discharge day two, she underwent a PET CT scan, which did not reveal any regional or metastatic disease (Figure 3), and on post-discharge day four, she began a nine-week course of chemoradiation with cisplatin. A follow-up PET CT performed one month after chemoradiation showed near complete metabolic response with marked decrease in mass burden. She then underwent successful endoscopic sinus surgery for tumor debulking. Two years later, she continues to receive routine surveillance PET CT scans, with her most recent study showing stable residual unresectable disease without evidence of metastasis. She reports hyposmia and xerostomia ever since oncologic treatment, but her visual acuity and intraocular pressure have progressively improved and are now within normal limits. She reports overall satisfaction with her vision.

## Discussion

In this case, we encountered a patient whose initial presentation was most concerning for an infectious or inflammatory process but was ultimately found to have an underlying neoplastic cause. According to a literature review by Badakere et al., neoplastic etiologies have been described in only a few case reports of OAS [2]. Primary head and neck tumors and hematologic malignancies, as well as metastatic lesions comprise the majority of neoplasms associated with OAS [2].

For instance, a case series by Prado-Ribeiro et al. described four male patients with known cancer; two patients had nasopharyngeal carcinoma, one had laryngeal squamous cell carcinoma, and one had squamous cell carcinoma of the tongue [2,3]. Two patients had loco-regional metastasis, and two had distant metastatic disease. All patients had a significant history of tobacco and/or alcohol use, which are major risk factors for head and neck cancer [3]. The most common presenting complaint was diplopia and ophthalmoplegia [2,3]. All patients received a combination of palliative chemotherapy and radiation with surgical debulking. All four patients died between nine and 11 months from the time of diagnosis of OAS [2,3]. Based on this, Ribeiro et al. concluded that OAS in a patient with head and neck cancer is an indicator of poor prognosis [2,3].

In this case, the patient was found to have OAS secondary to SMARCB1-deficient sinonasal carcinoma of the left paranasal sinus. Agaimy et al. described a case series of 39 patients with SMARCB1-deficient sinonasal carcinomas collected from multiple medical centers [4]. In their study, the tumors affected 23 males and 16 females with a median age of 52 years [4]. All patients presented with locally advanced disease. Thirty-one patients received surgery and/or chemoradiotherapy with curative intent [4]. At follow-up, 56% of patients had died at a median time of 15 months after initial diagnosis, also suggesting that SMARCB1-deficient sinonasal carcinoma is a rare but aggressive neoplastic entity rendering a generally poor prognosis [4].

Despite such a prognosis, however, the aforementioned patient in our report continues to live today, 29 months after her diagnosis, and has a near-normal ocular function. We believe that this positive outcome was made possible by a thorough emergency department evaluation followed by aggressive initiation of oncologic treatment. A few clinical learning points were gleaned from this case. First, the performance of nasal endoscopy in the ED, which led to the discovery of an anterior nasal mass, increased the suspicion of an underlying malignancy and expedited tissue collection. We believe endoscopy should be considered in cases of OAS to evaluate for such structural lesions in addition to other methods of advanced imaging. Additionally, in our case, the patient showed minimal clinical improvement in the hospital despite receiving multiple doses of broad-spectrum antibiotics and IV steroids. We believe this critical point should serve as a reminder to all clinicians to re-consider the differential diagnoses if a patient does not respond to therapy. To this point, we recognize that empiric antifungal and antiviral therapy could have been considered as case reports of invasive aspergillosis, mucormycosis, and herpes zoster have been described in association with OAS [1,2,5].

## Conclusions

This case report highlights the importance of evaluation for a broad range of etiologies when encountering a patient with OAS. We believe this case report will add to the growing body of literature associating neoplastic disease, specifically SMARCB1-deficient sinonasal carcinoma with resultant OAS, and will serve as a model for emergency department evaluation and management of patients with this condition.

## References

[1] Pittner Pittner (2018). Orbital apex syndrome. Ame Aca Opth.

[2] Badakere A, Patil-Chhablani P (2019). Orbital apex syndrome: A review. Eye Brain.

[3] Prado-Ribeiro AC, Luiz AC, Montezuma MA, Mak MP, Santos-Silva AR, Brandão TB (2017). Orbital apex syndrome affecting head and neck cancer patients: A case series. Med Oral Patol Oral Cir Bucal.

[4] Agaimy A, Hartmann A, Antonescu CR (2017). SMARCB1 (INI-1)-deficient sinonasal carcinoma: A series of 39 cases expanding the morphologic and clinicopathologic spectrum of a recently described entity. Am J Surg Pathol.

[5] Singh H, Kandel R, Nisar S, Das CJ, Dey AB (2014). An unexpected cause of orbital apex syndrome in an immune-competent elderly male. Oxf Med Case Reports.

